# Exosomes as drug delivery system in gastrointestinal cancer

**DOI:** 10.3389/fonc.2022.1101823

**Published:** 2023-01-25

**Authors:** Fangyuan Xie, Yueying Huang, Yangyang Zhan, Leilei Bao

**Affiliations:** Department of Pharmacy, Third Affiliated Hospital of Naval Medical University, Shanghai, China

**Keywords:** gastrointestinal cancer, exosomes, extracellular vesicles, drug delivery system, cancer therapy

## Abstract

Gastrointestinal cancer is one of the most common malignancies with relatively high morbidity and mortality. Exosomes are nanosized extracellular vesicles derived from most cells and widely distributed in body fluids. They are natural endogenous nanocarriers with low immunogenicity, high biocompatibility, and natural targeting, and can transport lipids, proteins, DNA, and RNA. Exosomes contain DNA, RNA, proteins, lipids, and other bioactive components, which can play a role in information transmission and regulation of cellular physiological and pathological processes during the progression of gastrointestinal cancer. In this paper, the role of exosomes in gastrointestinal cancers is briefly reviewed, with emphasis on the application of exosomes as drug delivery systems for gastrointestinal cancers. Finally, the challenges faced by exosome-based drug delivery systems are discussed.

## 1 Introduction

Gastrointestinal cancer is one of the most common and aggressive malignancies with relatively high morbidity and mortality in the world (1). According to the statistics of the International Agency for Research on Cancer (IARC), gastrointestinal cancer accounts for 45% of cancer-related deaths in China. At present, the therapeutic strategies for gastrointestinal cancer mainly include surgery, endoscopy, radiotherapy, chemotherapy, targeted therapy, and immunotherapy (2, 3). However, the prognosis of gastrointestinal cancer is still poor because early symptoms are occultic or asymptomatic and are detected at an advanced stage. Therefore, promising therapeutic strategies are needed to reduce the mortality of gastrointestinal cancer.

Recently, numerous studies have demonstrated that exosomes can be used as drug delivery systems and have the potential to improve the therapeutic effect on tumors (4–7). Exosomes are nanosized extracellular vesicles with a diameter of 40 - 100 nm, composed mainly of lipids, proteins, and genetic material. They are secreted by a variety of cells and widely distributed in body fluids, so they are biosafe, stable, and have good targeting specificity (8). In addition, exosomes as nanocarriers have the advantages of small size, negative charge, immune escape, and deep tissue penetration (9). Therefore, exosomes can be used as ideal natural nanocarriers for drug delivery. In this review, we briefly outline the role of exosomes in gastrointestinal cancers, focusing on the potential and challenges of exosomes as drug delivery systems for gastrointestinal cancers.

## 2 The role of exosomes in gastrointestinal cancers

**Figure 1:**
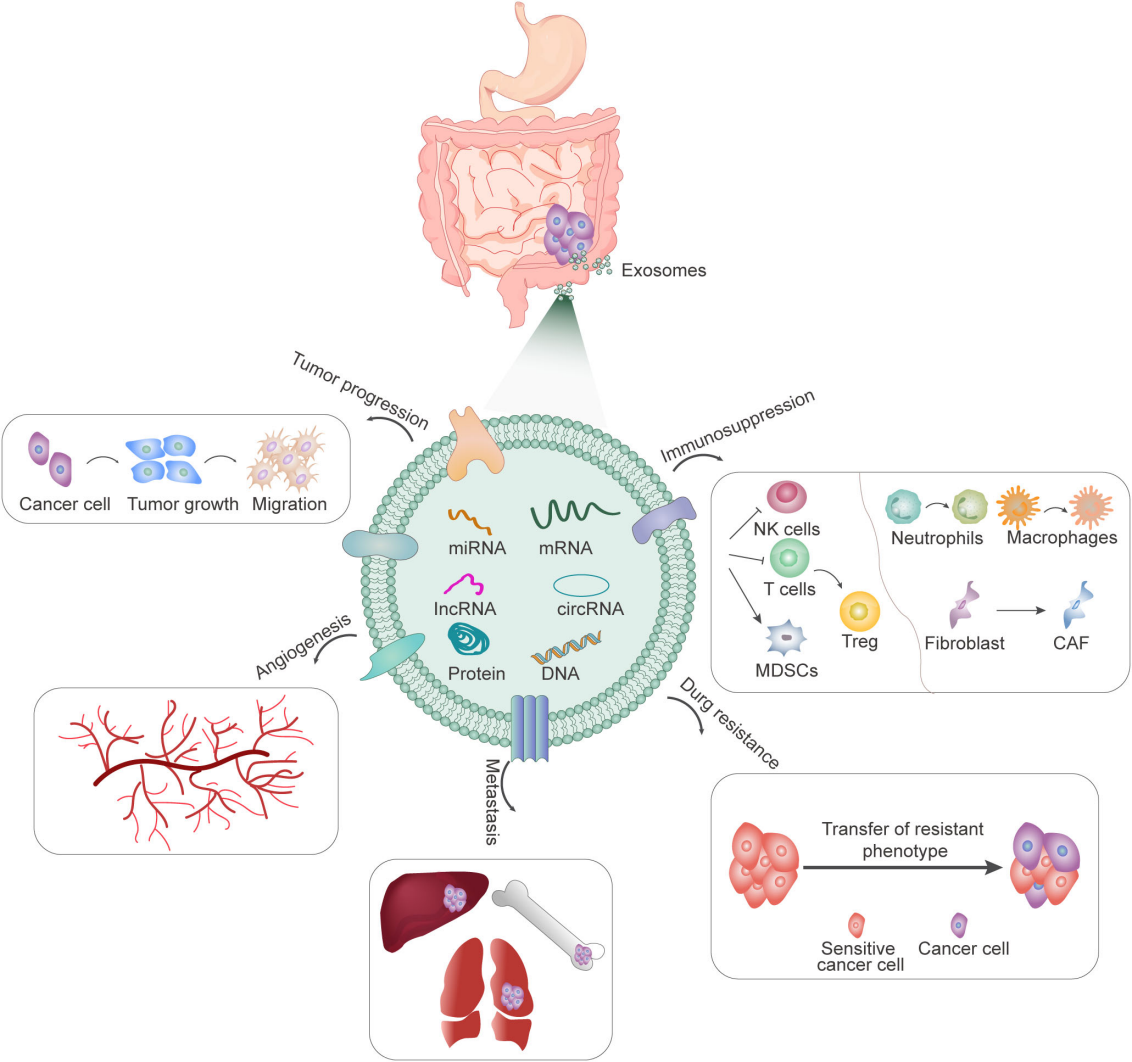
The role of exosomes in gastrointestinal cancers.

Exosomes may be involved in multiple processes of gastrointestinal progression, including proliferation, invasion and metastasis, angiogenesis, drug resistance, and immune escape (**Figure 1**). In 2009, it was first reported that gastric cancer cell-derived exosomes can promote the proliferation of SGC7901 and BGC823 cells through PI3K/AKT and MAPK/ERK activation (10). Subsequently, numerous studies have found that not only exosomes produced by tumor cells, but also exosomes secreted by mesenchymal stem cells (MSCs), fibroblasts, and other cells can also release contents to regulate the proliferation and metastasis of gastrointestinal cancers (11, 12). Exosomes can promote epithelial-mesenchymal transformation (EMT), improve the invasion and metastasis ability of receptor cells, and participate in matrix remodeling and metastasis formation. The important role of exosomes in gastrointestinal cancer metastasis is also manifested by their involvement in angiogenesis. Tumor cell-derived exosomes can promote angiogenesis and tumor progression in many ways (13, 14). Tumor-induced increased vascular permeability and angiogenesis are also important features of the formation of pre-metastatic niches, which in turn are closely related to distant metastasis of tumors. In addition, exosomes are involved in drug resistance, mediating drug resistance transfer between resistant and sensitive cells, as well as between tumor and stromal cells (15–18). Another important feature of gastrointestinal cancer-derived exosomes is their ability to modulate tumor immunity. Exosomes from different tumors carry different substances and information. They are specific to tumor cells and contain a variety of immunosuppressive molecules, which can inhibit the activity of NK cells and T cells, transform T cells into Treg-like cells, transform the phenotype of neutrophils and macrophages, promote the transformation of fibroblasts into cancer-associated fibroblasts (CAFs), and induce the proliferation of myeloid-derived suppressor cells (MDSCs), and play an important role in the suppression of tumor immune response (19, 20). In conclusion, tumor-derived exosomes contain a variety of proteins and miRNA, which bind to different targets to induce immunosuppression, forming a pre-metastasis microenvironment, and promoting tumor growth, differentiation, invasion, and metastasis.

## 3 Exosomes as drug delivery systems for the treatment of gastrointestinal cancers

Exosomes, as natural drug carriers, have been widely used and studied. It has many advantages over traditional nanocarriers in terms of drug and gene delivery. First, exosome delivery can improve the stability of drugs. For example, exosomes can protect nucleic acids from degradation during transport (21, 22). At the same time, exosomes can directly enter the cell fluid to avoid metabolic elimination, thus extending the drug circulation time. Second, exosomes have natural targeting capabilities based on parental cells. As drug delivery carriers, exosomes can target specific cell types and are suitable for targeted therapy. Moreover, exosomes from different cell sources express different molecules on the surface, so they have certain selectivity to the recipient cells, and thus are more advantageous in therapy (23). In addition, exosomes are nanoscale molecules that carry cell surface substances, so they have a strong ability to penetrate various biological barriers (24). Therefore, exosomes have a good application prospect in the field of drug carriers. Next, we will focus on the application of exosomes as drug delivery systems in gastrointestinal cancers (**Table 1**).

**Table 1:** Exosomes as drug delivery systems for the treatment of gastrointestinal cancers.

Function	Cancer type	Exosomes source	Engineering	Loading content	Ref
Inhibit tumor proliferation and metastasis	Gastric cancer	HER2-positive cancer cells	None	T-DM1	(25)
		U937 macrophages	None	miR-21 inhibitor	(26)
		HEK 293T cells	None	HGF siRNA	(27)
	Liver cancer	HEK 293T cells	Apo-A1	miR-26a	(28)
	Colorectal cancer	Human umbilical cord MSCs	None	miR-3940-5p	(29)
		Bone marrow MSCs	None	miR-34a-5p	(30)
		Human cord blood MSCs	iRGD-Lamp2b	anti-miRNA-221	(31)
		Milk	GE11 peptide	Oxaliplatin	(32)
		HEK 293 cells	AS1411 aptamer	Doxorubicin	(33)
		Bone marrow MSCs	MUC1 aptamer	Doxorubicin	(34)
		A33-positive human colorectal cancer cells	A33 antibody	Doxorubicin	(35)
	Pancreatic cancer	Human umbilical cord MSCs	None	miR-145-5p	(36)
		Pancreatic cancer cells	None	siPAK4	(37)
		Pancreatic cancer cells	RGD	Paclitaxel	(38)
Overcome drug resistance	Gastric cancer	HEK 293T cells	None	anti-miR-214	(39)
		HEK 293T cells	None	si-c-Met	(40)
	Colorectal cancer	HEK 293T cells	HER2-LAMP2	5-FU and miR-21 inhibitor oligonucleotide	(41)
		HEK 293T cells	None	si-ciRS-122	(16)
		FHC cell	None	miR-1915-3p	(42)
		FHC cells	None	Circular RNA FBXW7	(43)
		HEK 293T cells	None	Oxaliplatin and PGM5-AS1	(44)
	Pancreatic cancer	Melanoma cells	None	Survivin T34A	(45)
		Bone marrow MSCs	None	Paclitaxel and gemcitabine monophosphate	(46)
	Liver cancer	Adipose tissue-derived MSCs	None	miR-122	(47)
		Adipose tissue-derived MSCs	None	miR-199a	(48)
Immunotherapy	Colorectal cancer	Colorectal cancer cells	None	miR-34a	(49)
		M1-like macrophage	None	Zinc phthalocyanine	(50)
	Pancreatic cancer	Immunogenically dying tumor cells	MART-1 peptide	CCL22 siRNA	(51)
		Pancreatic cancer cells	None	Dendritic cells	(52)
		Bone marrow MSCs	None	Oxaliplatin prodrugs and galectin-9 siRNA	(53)
	Liver cancer	Dendritic cells	None	Alpha-fetoprotein	(54)

### 3.1 Exosomes-based drug delivery systems inhibit tumor proliferation and metastasis in gastrointestinal cancers

Exosomes are natural nanocarriers containing many active components. Therefore, they can be used to deliver a variety of components, including proteins, nucleic acids, and small molecules drugs. Trastuzumab emtansine (T-DM1) is an antibody-drug-conjugates (ADC) that binds the tubulin inhibitor emtansine to trastuzumab. The drug targets HER2-positive tumor cells and induces mitotic arrest and apoptosis through the intracellular release of the cytotoxic drug emtansine. It was found that exosomes derived from HER2^+^ cancer cells delivered T-DM1 to cancer cells and induce apoptosis (25).

miRNA is a class of highly conserved endogenous non-coding single-stranded small RNA that plays a vital role in gene regulation and tumor development. However, the successful delivery of miRNA is hampered by the difficulty of developing sustainable and efficient delivery systems. miRNA-21, one of the earliest miRNAs found in human cells, is highly expressed in a variety of cancers such as gastric cancer, and is closely related to the incidence of cancer. It has shown that exosomes derived from macrophages can be used as vectors to deliver exogenous miR-21 inhibitors into BGC-823 gastric cancer cells and regulate their proliferation (26). Moreover, exosome-mediated miR-21 inhibitor delivery has less cytotoxicity and more effective inhibition than conventional transfection methods. Liang et al. used engineered exosomes to target miR-26a to liver cancer cells expressing scavenger receptor class B type 1, down-regulating Cyclin D2, Cyclin E2, and CDK6 levels, inducing cell cycle arrest and inhibiting cell proliferation and metastasis in hepatocellular carcinoma (28). MSCs are ideal sources of exosomes for drug delivery because they are easy to isolate, have self-repair and multidirectional differentiation capabilities, as well as immune and specific homing properties. miR-3940-5p was significantly down-regulated in colorectal cancer. When it was loaded into MSCs derived exosomes and transfected into colorectal cancer cells, it inhibited EMT and invasion *in vitro* and inhibited tumor growth and metastasis *in vivo* (29). Similarly, MSCs-derived exosomes transfected with miR-34a-5p suppressed the growth of colorectal cancer cells and the tumorigenesis of colorectal cancer (30). In addition, functionalizing exosomes with targeting molecules can effectively enhance tumor-targeting ability. The anti-miRNA-221 oligonucleotide was delivered by human cord blood MSCs-derived exosomes, which were modified by the fusion gene iRGD-Lamp2b and were specifically taken up by tumor cells through their interaction with NRP-1 protein. The modified exosomes were significantly enriched at tumor sites and could significantly inhibit tumor growth both *in vitro* and *in vivo* (31). Human umbilical cord MSCs-derived exosomes can also effectively deliver miR-145-5p to pancreatic ductal adenocarcinoma cells, inhibits cell proliferation and invasion, and reduce tumor growth (36).

RNA interference has emerged as a promising clinical therapeutic tool that can lead to specific gene silencing. However, there are some limitations in the application of siRNA, including its poor cellular uptake and degradation by nucleases. Many vectors, such as viral vectors and cationic liposomes, have been used to deliver siRNA, but all have some limitations. Studies have shown that cell-derived exosomes are effective carriers of siRNA and can effectively inhibit tumor growth and angiogenesis in gastric cancer by delivering HGF siRNA (27). SiRNA (siPAK4) is encapsulated into pancreatic cancer-derived exosomes by electroporation for pancreatic ductal adenocarcinoma therapy. It can induce obvious tissue apoptosis and prolong the survival time of tumor-bearing mice (37). Pancreatic cancer-derived exosomes as an *in vivo* RNAi transfection agent showed efficacy comparable to that of polyethyleneimine (PEI), a commercial transfection agent, but was safer.

In addition to proteins and nucleic acids, exosomes can also be used to deliver small-molecule drugs, increase the stability and prolong the circulation time, thus improving the efficacy of drugs. To enhance the therapeutic effect, reduce the toxicity to normal cells, and expand the targeted drug delivery ability of exosomes, targeted modifications were made to endow them with cell and tissue specificity. Extracellular vesicles containing oxaliplatin bound to the GE11 peptide inhibit EGFR-expressing cancers through GE11 peptide-mediated EGFR targeting anticancer drug delivery. It showed that the engineered extracellular vesicles have the greatest therapeutic effect on tumor progression in colorectal cancer (32). Moreover, RGD and magnetic nanoparticles were conjugated to the surface of extracellular vesicles derived from human pancreatic cancer cells and loaded with paclitaxel for pancreatic cancer therapy. It can effectively penetrate and internalize tumor cells, and eventually cause tumor regression (38). Aptamer is an oligonucleotide sequence that can bind to target molecules with high affinity and specificity. It has the advantages of a wide range of target molecules, high stability, safety and economy, and simple preparation methods. Doxorubicin-loaded exosomes derived from HEK293 can target colorectal cancer by modifying with AS1411 aptamer (33). MSCs-derived exosomes loaded with doxorubicin can effectively target colorectal cancer and significantly inhibit tumor growth by covalently modifying carboxylic acid-end MUC1 aptamers (34). In recent years, the application of superparamagnetic nanoparticles in tumor therapy has attracted extensive attention. Exosomes were isolated from A33-positive human colorectal cancer cells and loaded with doxorubicin. Then, surface carboxylated superparamagnetic iron oxide nanoparticles coated with A33 antibodies bind to A33-positive exosomes and target A33-positive colorectal cancer cells. The results showed that A33 antibody-functionalized exosomes had the obvious tumor-targeting ability and have been confirmed to inhibit tumor growth (35). In conclusion, target-modified functional exosomes have proved to be novel and effective targeted drug delivery systems for gastrointestinal cancer therapy.

### 3.2 Exosomes-based drug delivery systems overcome drug resistance in gastrointestinal cancers

Drug resistance in tumor cells is usually identified as intrinsic (or innate) and extrinsic (or acquired) resistance. The former refers to cancer cells that are not sensitive to drugs at the beginning of treatment, while the latter refers to cancer cells that are originally sensitive to drugs, which developed drug resistance after repeated treatment and exposure to drugs. Once the tumor develops drug resistance, the drug cannot play an anti-cancer role. Even if the majority of the tumor is killed, the small number of drug-resistant cancer cells will continue to grow, causing cancer recurrence and rendering future anti-cancer chemotherapy ineffective. Therefore, drug resistance of cancer cells is one of the major challenges in cancer therapy. There are many factors leading to drug resistance, including abnormal gene expression, overexpression of transporters such as P-glycoprotein, and metabolic detoxification (55). However, the use of nanocarriers (such as exosomes) to deliver drugs can effectively overcome these factors, reverse drug resistance, and then exert good antitumor activity.

Cisplatin is an anti-cancer drug, which is widely used to treat a variety of cancers, including gastric cancer, colorectal cancer, and lung cancer. It inhibits tumor cell proliferation and induces apoptosis mainly by targeting DNA replication (56, 57). Although cisplatin has extensive anticancer activity, its use is limited due to its drug resistance and toxicity to untargeted tissues. The molecular mechanism of cisplatin resistance is complex and is mainly related to the abnormal expression of transporters, blocked apoptosis, enhanced intracellular detoxification, and enhanced DNA damage repair ability, as well as genetic and epigenetic changes (58, 59). miR-214 is an essential molecule in the process of drug resistance and is overexpressed in many malignant tumors. Exosomes containing anti-miR-214 can reverse cisplatin resistance and inhibit tumor growth in gastric cancer (39). In addition, c-MET, also known as hepatocyte growth factor receptor (HGFR), a protein with tyrosine kinase activity, is abnormally expressed or mutated in a variety of solid tumors and plays an important role in tumor proliferation, invasion, and metastasis. Transfecting HEK293T cells with si-c-Met and isolating exosomes can reverse the cisplatin resistance in gastric cancer, and inhibit the invasion and migration of gastric cancer cells and tumor growth (40).

5-fluorouracil (5-FU) is a first-line standardized chemotherapeutic drug for colorectal cancer, and the acquisition of 5-FU resistance often affects the therapeutic efficiency (60, 61). Exosome-delivered circ_0000338 enhances 5-FU resistance in colorectal cancer by negatively regulating miR-217 and miR-485-3p (62). Functional exosomes have been used to overcome drug resistance. Her2, a specific tumor-homing polypeptide, fuses with LAMP2, a protein found abundantly in exosome membranes. The HER2-LAMP2 fusion protein is expressed on the exosome surface and promotes the uptake of targeted cells through EGFR receptor-mediated endocytosis, effectively targeting colon cancer-resistant cells. The results showed that engineered exosomes loaded with 5-FU and miR-21 inhibitor oligonucleotides could effectively reverse colorectal cancer resistance and improve cancer therapeutic efficacy (41).

In addition, oxaliplatin-based chemotherapy is also one of the effective strategies for the therapy of colorectal cancer. Similarly, oxaliplatin resistance appears significantly in colorectal cancer, leading to treatment failure (63). Oxaliplatin-resistant colorectal cancer cells transfer ciRS-122 to oxaliplatin-sensitive cells *via* exosomes, thereby enhancing glycolysis and drug resistance. Exosome delivery of ciRS-122 siRNA enhances drug response (16). EMT refers to the transformation of epithelial cells into mesenchymal cells. The series of changes that occur after EMT activation contributes to the spread, invasion of surrounding tissues, and distant metastasis of tumor cells. EMT plays a key role in tumor invasion, metastasis, and drug resistance (64–66). More and more studies have shown that EMT markers can be used as prognostic indicators and potential therapeutic targets for colorectal cancer (67, 68). Exosome delivery of miR-1915-3p can downregulate the EMT-promoting oncogenes PFKFB3 and USP2, thereby improving the chemotherapeutic efficacy of oxaliplatin in colorectal cancer cells (42). Similarly, exosome delivery of circ-FBXW7 can inhibit EMT and oxaliplatin efflux by directly binding to miR-128-3p, increase oxaliplatin-induced apoptosis, and improve the sensitivity to oxaliplatin in colorectal cancer (43). Recently, the role of lncRNA in chemical resistance has been extensively studied. lncRNA PGM5 antisense RNA 1 (PGM5‐AS1) inhibits proliferation, migration, and acquired oxaliplatin tolerance in colon cancer cells. Exosomes co‐delivery of oxaliplatin and PGM5‐AS1 reverse drug resistance (44).

Gemcitabine is the current first-line treatment for pancreatic cancer. However, although gemcitabine has shown significant benefits in clinical application, its drug resistance severely limits its use. The transport, activation, and metabolism of gemcitabine are regulated by a variety of enzymes, and thus the development of resistance is regulated by a variety of factors (69). To overcome gemcitabine resistance in pancreatic cancer, survivin T34A was delivered by melanoma-cell-derived exosomes to restore the sensitivity of gemcitabine to pancreatic cancer cell lines. Compared with gemcitabine alone, apoptotic cell death is significantly increased (45). Moreover, exosomes from bone marrow-MSCs were used as homing carriers of pancreatic ductal adenocarcinoma to deliver paclitaxel and gemcitabine monophosphate as intermediates of gemcitabine metabolism. The results showed good penetration, anti-matrix, and anti-chemoresistance (46).

Exosome-based drug delivery systems have also shown promising therapeutic effects against drug resistance of other drugs in gastrointestinal cancers. miR-122 can promote the chemosensitivity of hepatocellular carcinoma cells. Delivery of miR-122 through adipose tissue-derived MSC exosomes can significantly improve the efficacy of sorafenib against hepatocellular carcinoma (47). Moreover, adipose tissue-derived MSC exosomes can effectively mediate the transfer of miR-199a to hepatocellular carcinoma cells and improve the sensitivity of hepatocellular carcinoma to doxorubicin (48). Taken together, this ability to deliver drugs and nucleic acids, along with other advantages such as low immunogenicity, biocompatibility, and natural targeting, make exosomes a promising and effective strategy for overcoming drug resistance in cancer therapy.

### 3.3 Exosomes-based drug delivery systems for immunotherapy in gastrointestinal cancers

Cancer immunotherapy is a therapeutic approach to control and eliminate tumors by modulating the immune system to activate anti-tumor immune responses or overcome tumor immune escape (70, 71). In recent years, the application of exosomes in cancer immunotherapy has been revealed. Numerous studies have shown that tumor and dendritic cells (DCs)-derived exosomes can express abundant tumor markers such as heat shock protein (HSP) and major histocompatibility complex (MHC). These molecules play a key role in antigen presentation and activation of T cells and have been demonstrated to provoke CD8^+^ T cell-mediated anti-tumor responses (72–75). Therefore, the application of exosomes in immunotherapy is of great significance to the progression of tumors, as the carrier of stimulating anti-tumor immune responses.

Tumor-derived exosomes are ideal antigen carriers, carrying many molecules and factors from tumor cells, and therefore easy to be recognized and taken up by immune cells. miR-34a is a major tumor suppressor that interferes with various colorectal cancer processes, including tumor proliferation, migration, and angiogenesis. Exosomes isolated from colorectal cancer cells and loaded with miR-34a mimic can reduce the expression of immune-evasion-related genes and induce cytotoxic T cells, significantly reducing the tumor size and prolonging the survival time of colorectal cancer mice (49). In addition, exosomes from immunogenically dying tumor cells modified with MART-1 peptide showed immunogenicity and were able to amplify CD8^+^ T cells for adoptive T cell therapy. The modified exosomes can enhance the anti-tumor immune response, and loaded with CCL22 siRNA can inhibit the expansion of Treg. The results showed that it was an effective preventive vaccine to delay tumor growth and a good adjuvant for pancreatic ductal adenocarcinoma chemotherapeutic drugs (51).

In addition to tumor-derived exosomes, DC-derived exosomes also have a promising application in tumor immunotherapy. DCs have unique antigen-presentation and activation properties of acquired and innate immune responses. DC-derived exosomes appear to act as antigen carriers, revealing their potential as cancer immunotherapy. Studies have shown that alpha-fetoprotein-rich DC-derived exosomes can induce effective antigen-specific anti-tumor immune responses and reshape the tumor microenvironment of hepatocellular carcinoma (54). Although DC-derived exosomes have a promising application prospect in tumor immunotherapy. However, the production of enough DC-derived exosomes remains a barrier to its widespread application in immunotherapy. Genetic engineering K562 has been used to produce artificial antigen-presenting cells, which secrete exosomes expressing HLA-A2 and costimulatory molecules that can enhance the anti-tumor immune effect of CD8^+^T cells, and these exosomes have a similar stimulative capacity as DC-derived exosomes (76).

Immunogenic cell death (ICD) is a kind of regulatory cell death, which can stimulate the immune system to produce immune responses through the release of tumor-associated antigens and tumor-specific antigens. It can be driven by different pressures, including intracellular pathogens, traditional chemotherapy drugs, targeted anti-cancer drugs, and a variety of physical therapies, such as radiotherapy and photodynamic therapy (77–79). The photosensitizer zinc phthalocyanine was added to exosomes from multiple cellular sources, such as immune cells, cancer cells, and external sources, to compare the antitumor effects of exosome-mediated photodynamic therapy. The results showed that M1-like macrophage-derived exosomes loaded with zinc phthalocyanine could initiate ICD, induce DC maturation, effectively inhibit colon cancer, and induce immune memory (50). These results indicate that the cell type and immune status from which exosomes are derived have a great influence on the therapeutic efficacy.

Cancer vaccines aim to stimulate the release and presentation of cancer antigens, immune cell initiation, and immune cell activation in the anti-cancer immune cycle. Once the immune cells are activated, they still need to complete the remaining steps: peripheral mobilization, infiltration to the tumor site, recognition of cancer cells, and activation of cytotoxicity against the cancer cell. Therefore, resistance mechanisms of anti-cancer immunity, especially in the tumor microenvironment, still reduce the efficiency of cancer vaccines, and to enhance cancer vaccines are being explored (80, 81). The combination of pancreatic cancer-derived exosome-loaded DCs vaccination with drugs that inhibit MDSCs, such as gemcitabine, sunitinib, and all-trans retinoic acid, can significantly inhibit the spread of metastasis, and prolong the survival time of mice due to the presence of more activated T cells in the tumor (52). Zhou et al. constructed a dual-delivery biosystem based on exosomes, which could significantly improve the tumor-targeting effect and thus increase the accumulation of drugs at the tumor site. This delivery system is composed of bone marrow MSC (BM-MSC) exosomes, surficially modified with oxaliplatin prodrugs and electroporation-loaded galectin-9 siRNA. The combination therapy can induce ICD in tumor cells, initiate DC maturation and antigen presentation, reverse immunosuppression, recruit anti-tumor cytotoxic T lymphocytes, and activate innate and adaptive anti-pancreatic ductal adenocarcinoma immunity (53).

## 4 Challenges for exosome-based drug delivery systems

As natural intercellular information carriers, exosomes have become one of the ideal drug delivery systems due to their nanoscale size, biocompatibility, permeability, and low immunogenicity. Although exosome-based drug delivery systems have been extensively studied, there still exist limitations in clinical application. First of all, it is difficult to obtain natural pure exosomes. Currently, exosomes are separated by a variety of methods, including ultracentrifugation, ultrafiltration, size-exclusion chromatography, polymer precipitation, immunoaffinity capture, and microfluidics-based techniques. Each method has advantages and limitations (8, 82). Ultracentrifugation is the current gold standard and the most commonly used exosome isolation approach. The resulting exosomes have a large volume but insufficient purity and the exosomes can be found to aggregate into blocks during electron microscopy identification, which is not conducive to subsequent experiments. Ultrafiltration is relatively simple and time-saving and is mostly used for the separation of exosomal RNA. However, exosomes may block the filtration pore, resulting in a shortened membrane life and low separation efficiency. Size-exclusion chromatography can obtain exosomes with high purity to ensure their integrity and activity. However, it is not suitable for amplification and only suitable for medium sample processing capacity. Polymer precipitation is simple and fast, but there are false positives (impurity protein or polymer), and mechanical forces or chemical additions will destroy exosomes. The immunoaffinity capture has the advantages of high specificity, simple operation, and no influence on the morphological integrity of exosomes. However, it has low efficiency and the biological activity of exosomes is easily affected by pH and salt concentration, which is not conducive to downstream experiments and difficult to be widely popularized. Microfluidics-based techniques are easy to automate but lack standardized and large-scale clinical sample testing and methodological validation. At present, no method can fully meet the demand, not only to maintain the integrity, high yield, and purity of exosomes but also to control the quality of exosomes. Therefore, the production of GMP-grade medicinal exosomes remains a major challenge, and the continuous optimization of the process will take a long time. Secondly, some researchers believe that exosomes are vesicles secreted by autologous cells and have the natural targeting ability based on donor cells, which can avoid phagocytosis by the mononuclear phagocyte system (MPS). Moreover, the expression of CD47 on the surface of exosomes is also conducive to avoiding clearance of MPS and prolonging blood circulation time (83, 84). However, some studies have shown that exosomes have a certain natural targeting ability but are not strong, and are easily recognized and quickly absorbed by MPS after systematic administration (85–87). Therefore, further research should reveal whether the clearance of exosomes by MPS is actually due to their intrinsic properties or exogenous characteristics acquired from *in vitro* culture. At present, to avoid rapid clearance and improve the targeting of exosomes, researchers have made various modifications to the exosome surface. However, although targeted modification methods have made progress in experiments, the *in vivo* environment is complex, and it is uncertain whether the modified exosome still has the expected targeting ability after entering the body. Therefore, targeted modification of exosomes is still the key point to be overcome. In addition, whether the modified exosomes induce immune responses should be further investigated in the future. Next, compared with conventional nanocarriers, common exosome loading strategies (such as passive mixing, electroporation, and exogenous loading) usually have low loading efficiency (<30%). These and other factors present significant challenges to the large-scale manufacturing of exosomes for drug delivery that, if overcome, could be translated into nanomedicine. Finally, the selection of the cellular sources of exosomes is also very important. Exosomes from different cellular sources have different components, and their potential biological functions are also significantly different. For example, exosomes derived from tumor cells as drug delivery systems can be well-homed to the tumor site, but at the same time have the risk of promoting tumor growth and immunosuppression (88). Exosomes derived from macrophages have a good inflammatory tendency and can cross the blood-brain barrier for the therapy of brain diseases (89). Exosomes derived from DCs have good immune effects (90). Therefore, selecting the cellular sources of exosomes is the premise of achieving the best therapeutic effect.

## 5 Conclusions

As extracellular nanovesicles, exosomes can deliver bioactive substances such as miRNA, lncRNA, and protein between cells, playing an extremely important role in the occurrence and development of gastrointestinal cancers. In addition, it can be used as a drug delivery system for the transportation of tumor therapeutic agents to inhibit tumor proliferation and metastasis, reverse drug resistance, and induce an anti-tumor immune response in gastrointestinal cancers. Although exosomes as drug delivery systems have potential advantages such as biocompatibility, stability, and intrinsic targeting, the research on exosomes as drug delivery systems is still insufficient, and many problems remain to be solved. The research aimed at clinical transformation should make continuous efforts to improve the production, purity, targeting, and bioactivity of exosomes. In conclusion, the clinical successful application of exosomes as drug delivery systems will take some time, but it is believed that it will benefit the majority of patients soon.

## Author contributions

FX and LB provided the direction and guidance for this manuscript. FX wrote the whole manuscript. YH, YZ, and LB revised this manuscript. All authors contributed to the article and approved the submitted version.
